# Sub-100 Femtosecond All-Optical Modulation Beyond Electron–Phonon Limits

**DOI:** 10.1007/s40820-026-02166-z

**Published:** 2026-04-07

**Authors:** Renxian Gao, Jiayu Li, Xiaoxiang Dong, Yonglin He, Wenbin Chen, Peiwen Ren, Xiaoyu Zhao, Ming-De Li, Zhilin Yang

**Affiliations:** 1 College of Physical Science and Technology, Xiamen University, Xiamen, 361005 People’s Republic of China; 2 College of Materials and Environmental Engineering, Hangzhou Dianzi University, Hangzhou, 310018 People’s Republic of China; 3 Key Laboratory for Preparation and Application of Ordered Structural Materials of Guangdong Province, Department of Chemistry, Shantou University, Shantou, 515063 People’s Republic of China; 4 School of Physics and Optoelectronic Engineering & Guangdong Provincial Key Laboratory of Sensing Physics and System Integration Applications, Guangdong University of Technology, Guangzhou, Guangdong 510006 People’s Republic of China

**Keywords:** Sub 100 fs all Optical modulation, Silver, Silicon metastructure; Interfacial hot, Carrier dynamics

## Abstract

Ultrafast all-optical modulators are central to the advancement of next-generation photonic computing and signal-processing systems. However, the intrinsic electron–phonon relaxation bottleneck in plasmonic materials has long constrained modulation speeds to the picosecond regime, hindering the realization of sub-100 fs modulation. Here, we report a metastructured silver–single-crystal silicon nanodisk antenna that delivers experimentally resolved sub-100 fs all-optical modulation. Distinct from conventional planar metal–semiconductor junctions, the nanodisk architecture spatially co-localizes plasmonic energy deposition with the metal–semiconductor transfer boundary within a nanoscale-confined volume. This configuration markedly shortens hot-carrier transport pathways and preferentially activates interfacial carrier extraction during the earliest relaxation stage, thereby establishing an interface-dominated modulation pathway that precedes electron–phonon thermalization. By enabling modulation on timescales comparable to intrinsic electronic response limits, this work establishes a physical foundation for ultrafast photonic modulation, including femtosecond free-space photonic computing architectures, temporal optical gating, and other ultrafast systems constrained by carrier or cavity lifetimes.

**Supplementary Information:**

The online version contains supplementary material available at 10.1007/s40820-026-02166-z.

## Highlights

- Engineered Ag–Si interfacial plasmonic modes enable deconvolution-verified sub-100 fs all-optical modulation, overcoming the temporal bottleneck imposed by electron–phonon relaxation in conventional plasmonic structures.
- A unified electromagnetic–thermal physical model reveals an interface-governed nonthermal carrier extraction pathway faster than electron–electron and electron–phonon scattering, quantitatively explaining the observed sub-100 fs dynamics.

**Supplementary Information:**

The online version contains supplementary material available at 10.1007/s40820-026-02166-z.

## Graphical abstract

**Figure:**
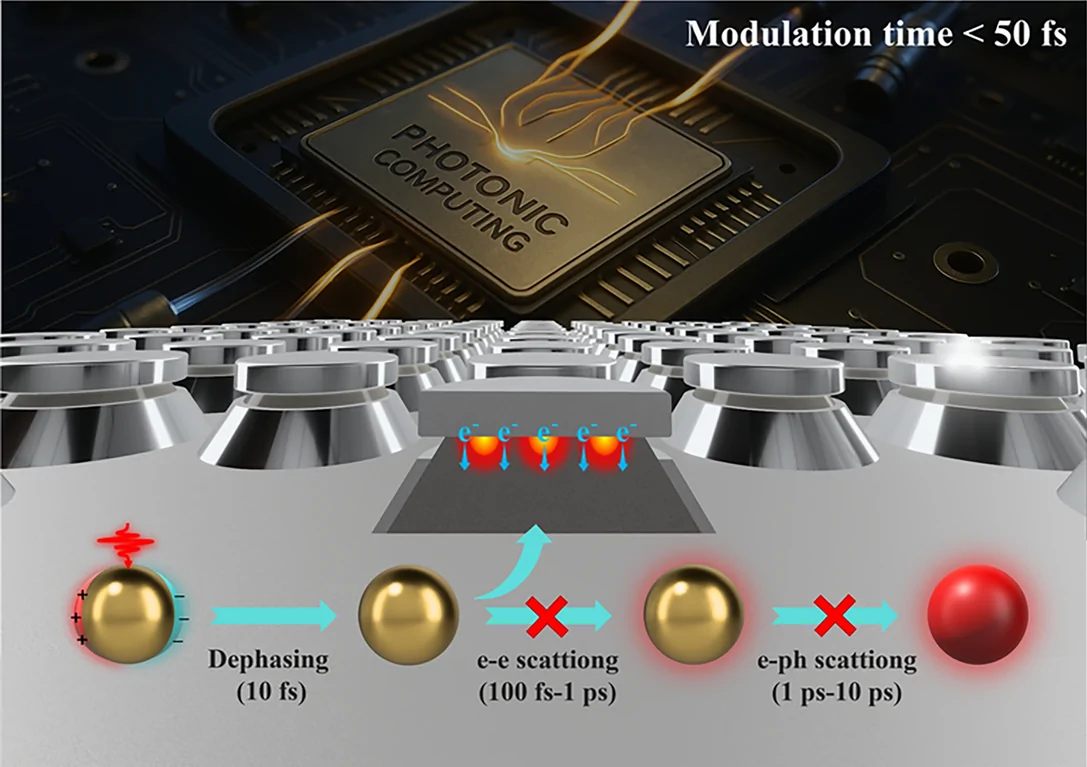

## Introduction

The ever-increasing demand for high-speed optical computing and all-optical signal processing has driven significant advancements in ultrafast optical modulators. These devices play a pivotal role in enhancing data throughput, energy efficiency, and integration density in next-generation photonic circuits [1–6]. Plasmonic all-optical modulators have emerged as promising candidates due to their ability to confine and manipulate light at deep-subwavelength scales, with ultrafast response times and strong potential for high-density photonic integration [7–9]. However, despite the theoretical potential of plasmonic systems, the intrinsic relaxation mechanisms associated with hot-carrier dynamics impose severe limitations on modulation speeds, constraining the realization of all-optical switching below 100 fs [7–10]. Overcoming these fundamental constraints demands innovative plasmonic structure designs that enable efficient carrier extraction and achieve ultrafast dynamics on the tens-of-femtoseconds timescale.

The modulation speed of plasmonic devices is inherently governed by the complex interplay of nonequilibrium carrier dynamics [11–13]. Upon photoexcitation, plasmonic systems exhibit a cascade of ultrafast processes, including plasmon dephasing and hot-electron generation, followed by electron–electron scattering within tens to hundreds of femtoseconds, electron–phonon thermalization over several picoseconds, and ultimately phonon dissipation into the surrounding environment on nanosecond timescales [14–16]. While electron–electron interactions contribute to rapid thermalization within the electronic subsystem, the subsequent coupling to the lattice introduces a bottleneck that significantly limits the recovery time of plasmonic modulators [17, 18]. In recent years, hot-carrier-assisted ultrafast modulation mechanisms have been extensively investigated in plasmonic metasurface and metal–semiconductor or metal–2D-material hybrid nanostructures [10, 12, 14, 19, 20]. In these systems, non-radiative plasmon decay generates nonequilibrium hot carriers within the metal, and interfacial extraction or energy transfer is employed to accelerate the onset of the transient optical response. However, despite the presence of a hundred-femtosecond fast component in many reported transient traces, the dynamics are typically followed by a pronounced sub-picosecond or picosecond relaxation tail governed by electron–phonon coupling and lattice heating. Such lattice-mediated thermo-optic contributions persist beyond the initial electronic excitation and ultimately constrain both the achievable modulation contrast and the recovery speed. Parallel efforts based on engineered nanocavities and nonlinear optical materials have enabled sub-picosecond switching by leveraging strong optical Kerr effects, yet the ability to achieve sub-100 fs reset times remains challenging due to the persistent influence of electron–phonon interactions [21–25].

**Fig. 1:**
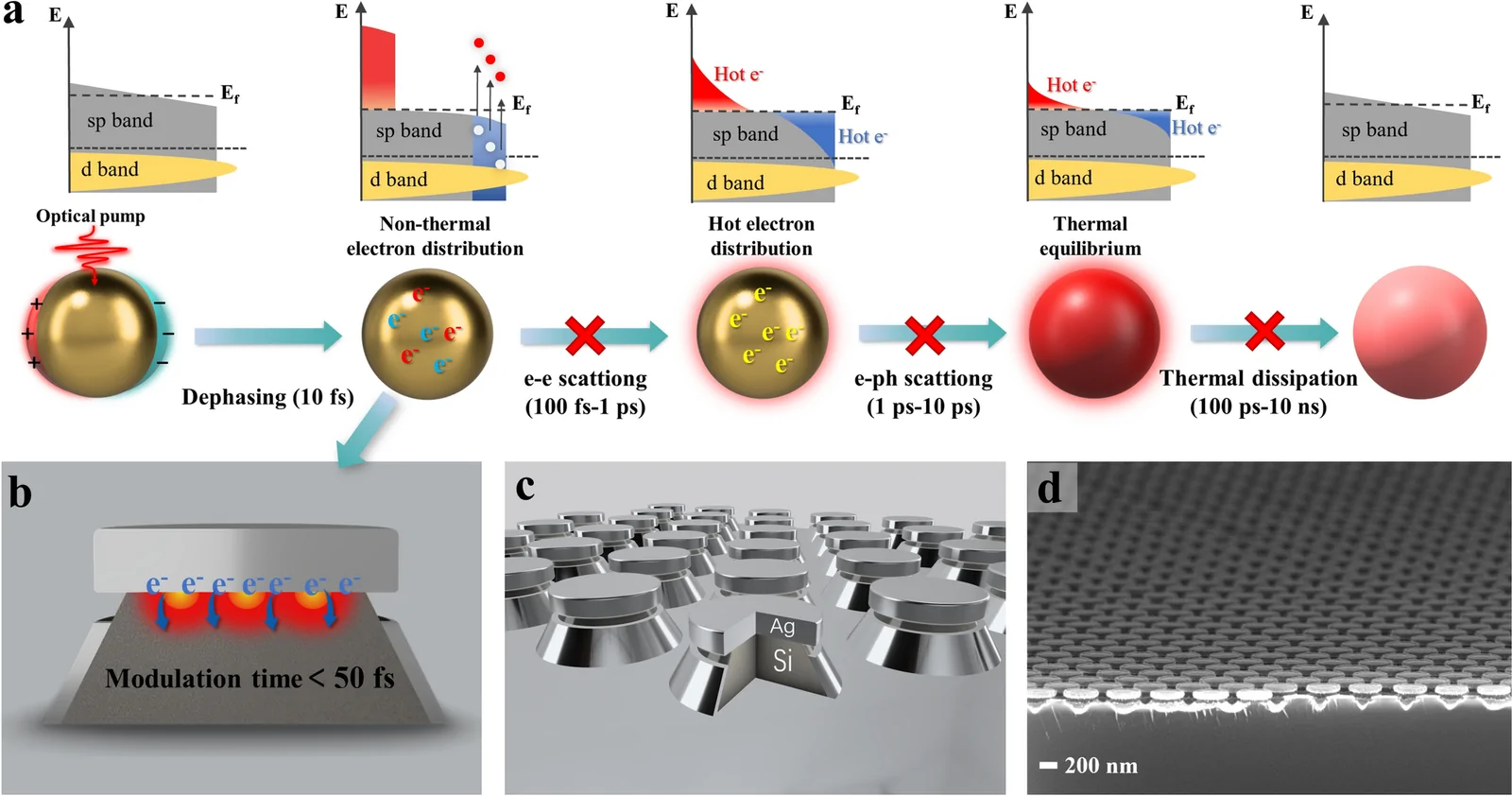
Schematic diagrams and scanning electron microscopy image. **a** Schematic diagram illustrating the dynamics of surface plasmon relaxation. **b** Schematic diagram illustrates the rapid lossless relaxation pathway of surface plasmon resonance-induced nonthermal equilibrium electrons in SSDMA. **c** Schematic diagram illustrates the structure of the SSDMA, highlighting the complex arrangement of single-crystal silicon nanodisks integrated with overlaid silver nanodisks. **d** Scanning electron microscopy image of the metastructure

To address these challenges, we introduce a metastructured nanophotonic antenna capable of sub-100 fs all-optical modulation, with the underlying concept and design principles illustrated in Fig. 1a, b. The device is built upon a silver–single-crystal silicon nanodisk metastructure antenna (SSDMA), which exploits the interfacial plasmonic confinement at the metal–semiconductor boundary to activate an ultrafast, nonthermal hot-electron transfer pathway. By precisely engineering the near-field distribution at the Ag-Si boundary, the structure spatially co-localizes plasmonic energy deposition with the carrier-transfer interface within a nanoscale-confined volume. This configuration markedly shortens hot-carrier transport pathways and preferentially activates interfacial carrier extraction during the earliest relaxation stage, enabling femtosecond-scale carrier transfer that competes with electronic thermalization and precedes substantial lattice heating. Consequently, the transient optical response is governed by an interface-dominated electronic pathway rather than lattice-mediated thermo-optic effects, allowing modulation to occur on timescales approaching intrinsic electronic response limits. Beyond demonstrating sub-100 fs all-optical modulation, this work establishes an interface-governed physical mechanism for overcoming electron–phonon-limited temporal constraints in plasmonic systems and provides a generalizable design framework for ultrafast photonic components operating near intrinsic carrier or cavity-lifetime limits.

## Experimental Section

### Materials

Single-crystal silicon wafers (⟨100⟩ orientation, resistivity 1–10 Ω cm) were obtained from Zhongsheng Silicon Technology Co., Ltd. (China). Polystyrene (PS) nanospheres with an initial diameter of 500 nm were purchased from Aladdin Biochemical Technology Co., Ltd. (China). High-purity silver pellets (99.99%) for electron-beam evaporation were supplied by Zhongnuo Advanced Material Co., Ltd. (China). High-temperature-resistant polyimide tape used for template removal was obtained from DuPont™ (USA). Gases used in the RIE processes, including argon (Ar, 99.999%), oxygen (O₂, 99.999%), and sulfur hexafluoride (SF₆, 99.99%), were provided by Hangzhou Jingong Gas Co., Ltd. (China). Deionized (DI) water (18.2 MΩ cm) was produced using a Milli-Q purification system. All chemicals and reagents used for substrate cleaning—acetone (ACS grade), isopropanol (IPA, ≥ 99.7%), and ethanol (≥ 99.5%)—were purchased from Sinopharm Chemical Reagent Co., Ltd. (China).

### Template-Assisted RIE Lithography for SSDMA Metastructure Fabrication

To achieve high-precision fabrication of metal–single-crystal-silicon interface localized-field metastructure arrays, we employed a rigorously controlled reactive-ion etching (RIE) workflow. The overall process is outlined in Fig. S1. A monolayer of hexagonally close-packed polystyrene (PS) nanospheres was first assembled at the air–water interface to serve as a soft lithographic template. The sphere diameter was subsequently reduced in an RIE system, enabling precise tuning of the structural pitch and aperture. The size-adjusted PS monolayer was then transferred onto the single-crystal silicon wafer and used as a mask in a second RIE etching step (Sentech RIE), during which selective anisotropic etching of the silicon produced a periodic nano-pyramidal topography beneath the sphere array. Progressive extension of the etching duration yielded well-defined and uniform single-crystal silicon nanopillars, as shown in step 2 of Fig. S1. After removing the PS spheres using high-temperature-resistant adhesive film, a clean silicon nanopillar array was obtained (step 3). Finally, the pre-patterned silicon template was introduced into an electron-beam evaporation system (Atemd-500), where a conformal silver nanofilm was deposited at a rate of 4 Å s⁻^1^ under high vacuum. Increasing the film thickness gradually decorated the silicon nanopillars with a continuous plasmonic coating, culminating in the formation of the SSDMA architecture.

## Results and Discussion

### Design Principle and Structural Characterizations

To achieve sub-100 fs ultrafast all-optical modulation across multiple discrete wavelengths, a metastructure array was carefully designed. This array included silver nanodisks placed on top of an array of monocrystalline silicon nanodisks (see Fig. 1b, c). The metastructure array with the metal-monocrystalline silicon interface was fabricated using an inductively coupled plasma etching technique, and further information is provided in Fig. S1. The structural morphology of the metasurface, as depicted in Fig. 1d, demonstrates a high level of conformity, with a schematic illustration shown in Fig. 1c. Specifically, the silicon nanodisk arrangement follows a hexagonal close-packed pattern with a periodicity of 500 nm, while the silver nanodisks exhibit a diameter of 300 nm. The distinctive taper design is characterized by a larger base diameter for the silicon nanotapers than their top diameter, facilitating partial encasement by the silver film. The total height of the nanotapers measures 250 nm, and the silver nanodisks have a thickness of 40 nm. Scanning electron microscopy (SEM) images of a sample from different viewing angles are included in Fig. S2.

### Steady-State Optical Response and Resonance Mode Analysis

**Fig. 2:**
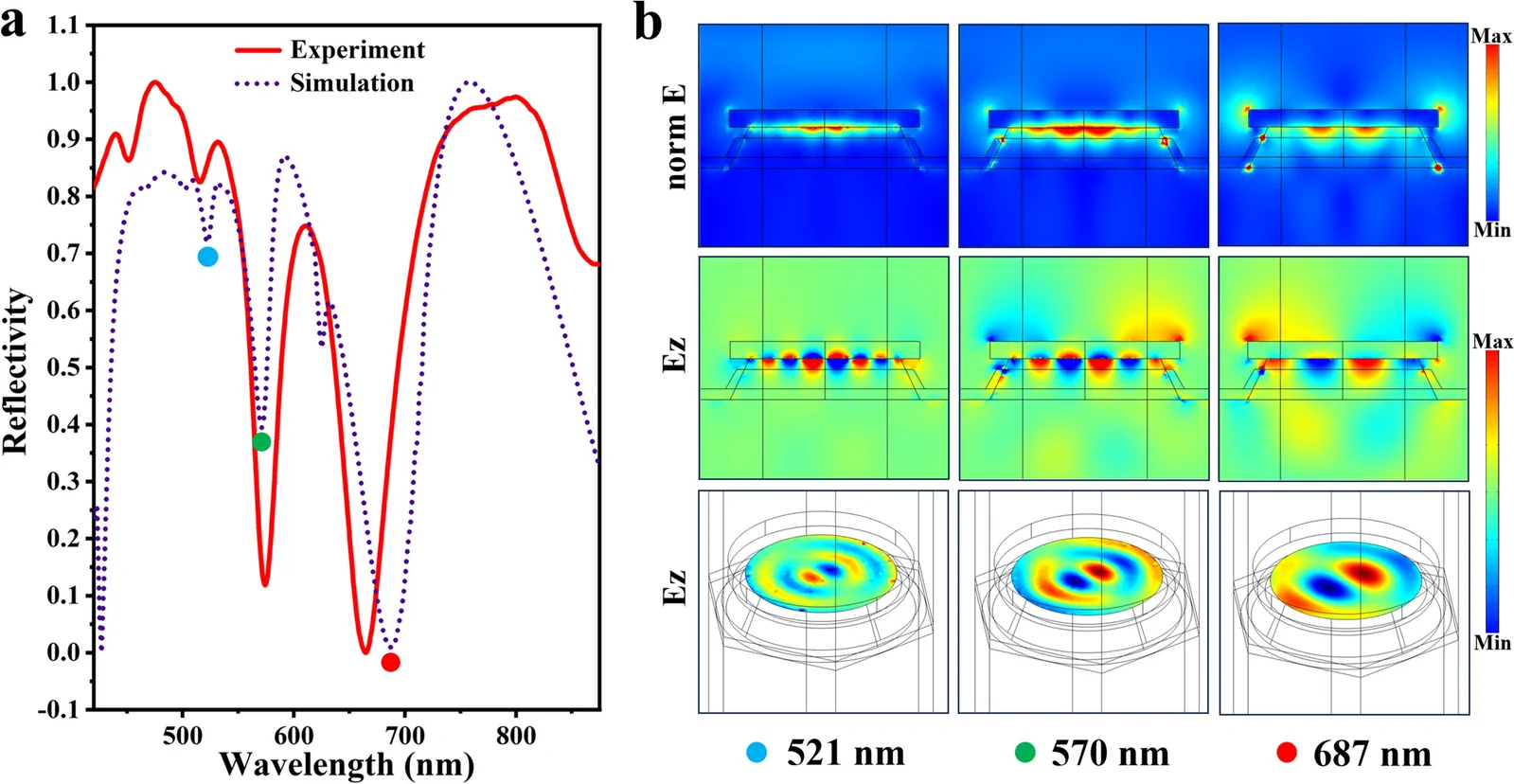
Steady-State Reflectance Spectrum, Numerical Simulation Spectrum, and Electric Field Distribution. **a** Reflectance spectrum of SSDMA arrays, with the continuous red line representing the measured experimental data and the purple dashed line indicating the corresponding results from finite element electromagnetic simulations. **b** Finite element analysis of electric field distributions induced by monochromatic planar waves. The simulation presents spatially resolved electric field intensities (E-field modulus) and the corresponding Z-component distributions for incident wavelengths of 521 nm, 570 nm, and 687 nm. The top row shows the E-field modulus across the simulation domain, highlighting peak field enhancements. The middle row displays the Z-component of the electric field, while the bottom row provides a three-dimensional view of the Z-component

Prior to transient optical characterization, we performed steady-state reflectance spectroscopy together with corresponding finite-element electromagnetic simulations of the SSDMA. As shown in Fig. 2a, the experimental reflectance spectrum (red solid line) exhibits good qualitative agreement with the simulated spectrum (purple dashed line). In the experimental data, three well-defined reflectance minima are observed at 514, 572, and 660 nm, with the deepest minimum at 660 nm and progressively weaker minima at shorter wavelengths. In the numerical simulation, the corresponding resonance dips are located at 521, 570, and 687 nm, respectively. The slight discrepancies between experiment and simulation mainly arise from unavoidable fabrication-related deviations and differences in optical constants between the fabricated structures and the idealized simulation model. To elucidate the near-field resonance modes associated with these dips, we simulated the localized electric-field distributions under normally incident plane waves at the corresponding wavelengths. The results (Fig. 2b) reveal distinct surface plasmon resonance modes, with the localized fields concentrated at the silver–silicon nanodisk interface. This spatial configuration of plasmonic hotspots is expected to shorten the relaxation pathway for hot-electron injection into single-crystal silicon, thereby enabling femtosecond-scale optical modulation. Subsequent transient optical measurements further test this hypothesis and provide direct experimental validation of the proposed mechanism. Moreover, the spatial arrangement of localized fields at resonant wavelengths on the silver–silicon interface offers a pathway toward multi-frequency ultrafast plasmon-driven all-optical modulation. To further substantiate the design of the SSDMA metastructure, we numerically compared the photoinduced electric-field intensity at the silver–silicon interface between the SSDMA and pure silver nanodisk arrays, as shown in Fig. S3.

### Femtosecond Pump–Probe Probing of Interface-Localized Plasmon Dynamics

To assess the transient all-optical modulation capabilities of the metastructure, we performed transient optical experiments utilizing a femtosecond pump-probe system. The experimental configuration included a pulsed laser with a primary wavelength of 800 nm and an 84 fs pulse duration. This laser could produce pump pulses at the specified wavelength following amplification through an optical parametric amplifier. The pump light was accurately directed onto the sample through a lens, leading to excitation with a power density of 51 μJ cm^−2^. Concurrently, a second beam was directed through a sapphire crystal to generate a super-continuum probe pulse that encompassed wavelengths between 430 and 830 nm. Upon interaction with the sample, the probing light was detected using a spectrometer. The acquired signals were then analyzed with differential circuitry, enabling a comprehensive evaluation of the sample response to transient optical modulation. The schematic of the transient pump-probe optical setup is shown in Fig. S4.

**Fig. 3:**
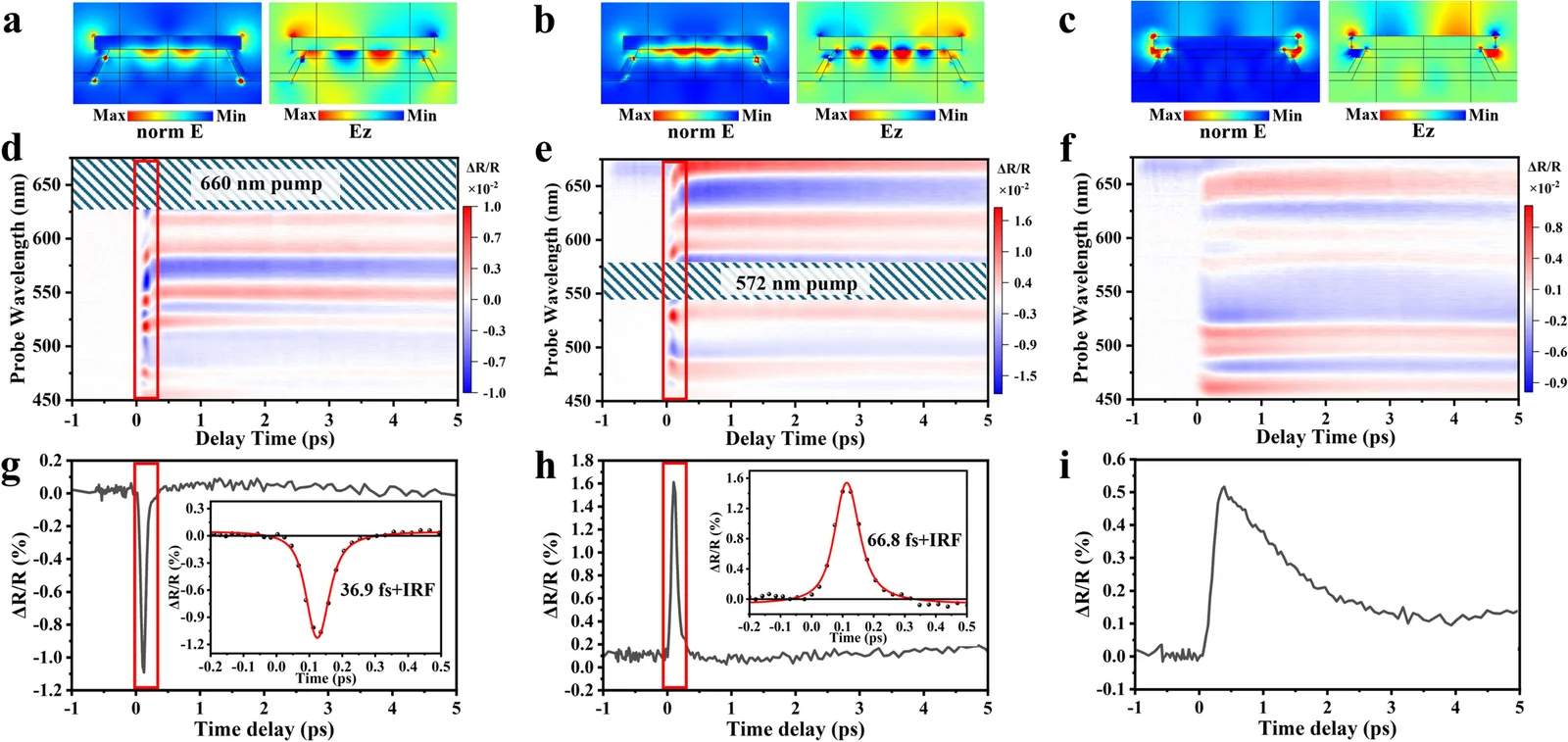
Transient optical measurement. **a-c** Near-field numerical simulation results of the sample corresponding to incident laser wavelengths of 660, 572, and 400 nm. **d-f** Transient reflection spectra of the sample in the range of 450–675 nm with pump laser wavelengths of 660, 572, and 400 nm, where the maximum delay time is 5 ps. **g** Transient dynamic spectral lines with a pump wavelength of 660 nm and a probe wavelength of 559 nm, the solid red line in the inset corresponds to a fit considering the convolution between IRF and the Lorentz function. **h** Transient dynamic spectral lines with a pump wavelength of 572 nm and a probe wavelength of 589 nm, the solid red line in the inset corresponds to a fit considering the convolution between IRF and the Lorentz function. **i** Transient dynamic spectral lines with a pump wavelength of 400 nm and a probe wavelength of 514 nm

Based on the near-field simulation results illustrated in Fig. 2b, the plasmonic electric fields generated by incident light with wavelengths of 572 and 660 nm are clearly primarily concentrated at the interface between silver and silicon. Therefore, these wavelengths were selected for pump pulses to induce transient excitation dynamics, in contrast to the 400 nm wavelength pump light that activates surface plasmon resonance modes located further from the silver-silicon interface. Figure 3a–c shows the numerical simulation results illustrating the distribution of the surface plasmon electric field at wavelengths of 572, 660, and 400 nm. Figure 3d–f displays the transient reflection spectra of the sample when excited by pulsed light at the wavelengths shown in Fig. 3a–c. Figure 3d, e reveals multiple ultrafast dynamic signals within the wavelength range of 450 to 700 nm. The observed signals are consistent with the previously discussed steady-state spectra, providing clear evidence of the modulation of the resonance absorption modes of the surface plasmon resonance at the silver-silicon interface for central wavelengths of 514, 572, and 660 nm. Figure 3d, e demonstrates distinct femtosecond fast component transient signals, as well as slower transient signals typically associated with nonequilibrium electron thermal diffusion induced by surface plasmons, with relaxation times at the picosecond level. To determine the source of the femtosecond fast component signals, a comparative analysis was conducted utilizing a 400 nm wavelength pump laser, as illustrated in Fig. 3f. The femtosecond fast component transient signals observed in Fig. 3d, e are lacking in this figure. Additionally, Fig. 3c reveals that when the sample is subjected to a 400 nm incident pump laser, the localized plasmonic field is predominantly restricted to the edges of the silver nanodisks, with hotspots located away from the silver-silicon interface. The findings from these comparative experiments confirm that the femtosecond fast components observed in Fig. 3d, e are related to the ultrafast dynamics of surface plasmons at the interface between silver and silicon.

The ultrafast dynamics of surface plasmon resonance typically span from a few femtoseconds to several hundred picoseconds, influencing the temperature and density of free carriers and phonons in metals and thereby modifying their dielectric function, which leads to pronounced nonlinear optical Kerr effects [26–28]. However, the rate of energy transfer from hot electrons to phonons in metals is intrinsically slow, restricting the modulation speed of conventional plasmonic nanostructures to the picosecond regime [29, 30]. To overcome this temporal bottleneck, we analyzed in detail how the spatial distribution of plasmonic modes governs the transient carrier dynamics. Comparing the hotspot distributions in Fig. 3a, b with the resonance-field localization in Fig. 3c reveals a strong correlation between the appearance of sub–100 fs transient components in Fig. 3d, e and the effective confinement of the plasmonic field at the silver–silicon interface. Specifically, the interface-localized plasmonic resonance mode promotes efficient Landau damping, generating high-energy nonequilibrium hot electrons [14–16]. Importantly, hot-carrier collection across a Schottky junction follows the established three-step picture (generation–transport–collection), and is constrained by both the energetic condition $E-{E}_{F}>{\Phi }_{B}$ and the interfacial transmission selectivity [17, 31–33]. For the Ag–Si junction, the Schottky barrier height lies in the sub-eV range (typically ${\Phi }_{B}\sim 0.6\hspace{0.17em}-\hspace{0.17em}0.8$ eV for n-type Si). Under visible excitation, the Landau-damping-generated nonthermal distribution contains a high-energy tail that can satisfy $E-{E}_{F}>{\Phi }_{B}$, making interfacial charge transfer into Si energetically accessible [34, 35]. Because the plasmonic hotspot is co-localized with the Ag–Si interface, the hot-carrier transport distance is minimized, allowing carriers to reach the boundary before substantial electron–phonon energy transfer develops and thereby enabling a sub-100 fs interfacial dielectric perturbation that precedes the conventional electron–phonon-mediated picosecond pathway. Consistent with this picture, all three resonance modes in Fig. 2b (514, 572, and 660 nm) exhibit strong Ag–Si interface localization, so excitation at either 572 or 660 nm can trigger an interface-mediated dielectric perturbation that simultaneously influences the interface-localized resonances, as manifested by the pronounced femtosecond components highlighted in Fig. 3d, e.

In contrast, when the plasmonic excitation is localized away from the Ag–Si interface (as in Fig. 3c), the generated hot electrons cannot efficiently transfer into the semiconductor. Instead, they dissipate energy through electron–electron and electron–phonon scattering within the metal, producing slower picosecond-scale relaxation dynamics (Fig. 3f, i). This scattering-dominated pathway fundamentally limits the achievable modulation speed. From a design perspective, the plasmonic configurations in Fig. 3a, b dramatically enhance the spatial overlap between near-field hotspots and the carrier-injection region. Such co-localization minimizes the transport distance of hot electrons and greatly improves injection efficiency, thereby enabling multi-wavelength, sub 100 fs modulation of the transient optical response.

To precisely ascertain the all-optical modulation speed of the sample, we analyzed the transient dynamics curves at probe wavelengths of 559 and 572 nm extracted from Fig. 3d and e, as depicted in Fig. 3g, h. To quantitatively extract the intrinsic ultrafast response, the measured transient signals were modeled as the convolution of an independently measured Gaussian instrument response function (IRF) and a single exponential sample response. The IRF width was fixed to the cross-correlation FWHM of 100.8 fs, ensuring that no instrument-dependent parameter was allowed to float during fitting. Under this rigorously constrained deconvolution procedure, the probe wavelength resolved dynamics yield characteristic time constants of 37 ± 9 and 67 ± 14 fs at 559 and 589 nm, respectively. The corresponding transient kinetic traces with error bars are presented in Fig. S5, and detailed fitting procedures and numerical validation are provided in Section S6. Because the fastest extracted timescale approaches the IRF width, it represents an IRF-conditioned estimate rather than a strictly resolved decay constant. The intrinsic response is therefore conservatively assigned to a sub-100-fs regime. Despite this limitation, the fits consistently show that the Ag–Si interfacial hot-electron-induced permittivity perturbation occurs well before electron–phonon thermalization, confirming sub-100 fs dynamics substantially faster than conventional lattice-mediated relaxation. In addition to the response time, we quantitatively evaluated the modulation depth and switching contrast. At a pump power density of 51 μJ cm^−2^, the peak transient reflectance changes $(\Delta R/R)_{peak}$ reach 1.1% and 1.6% at the 559 and 572 nm probe wavelengths, respectively (Fig. 3g, h). We then assessed the switching contrast using an on–off ratio metric. As shown in Fig. 2b, the maximum on–off ratio exceeds 100 within the first 5 ps after excitation. The on–off ratio is defined as:

1 $$ On{-}off~\;ratio = \frac{{{\mid }(\Delta R/R)_{{peak}} {\mid }}}{{{\mid }(\Delta R/R)_{{off}} {\mid }}}  $$

where $(\Delta R/R)_{off}$ is defined as the maximum residual modulation magnitude within the 0.5–5 ps delay window after the initial peak. To the best of our knowledge, this value is among the highest reported for plasmonic ultrafast all-optical modulation systems [12, 14, 19–21].

### Electron-Blocking Validation and Power-Dependent Ultrafast Dynamics

**Fig. 4:**
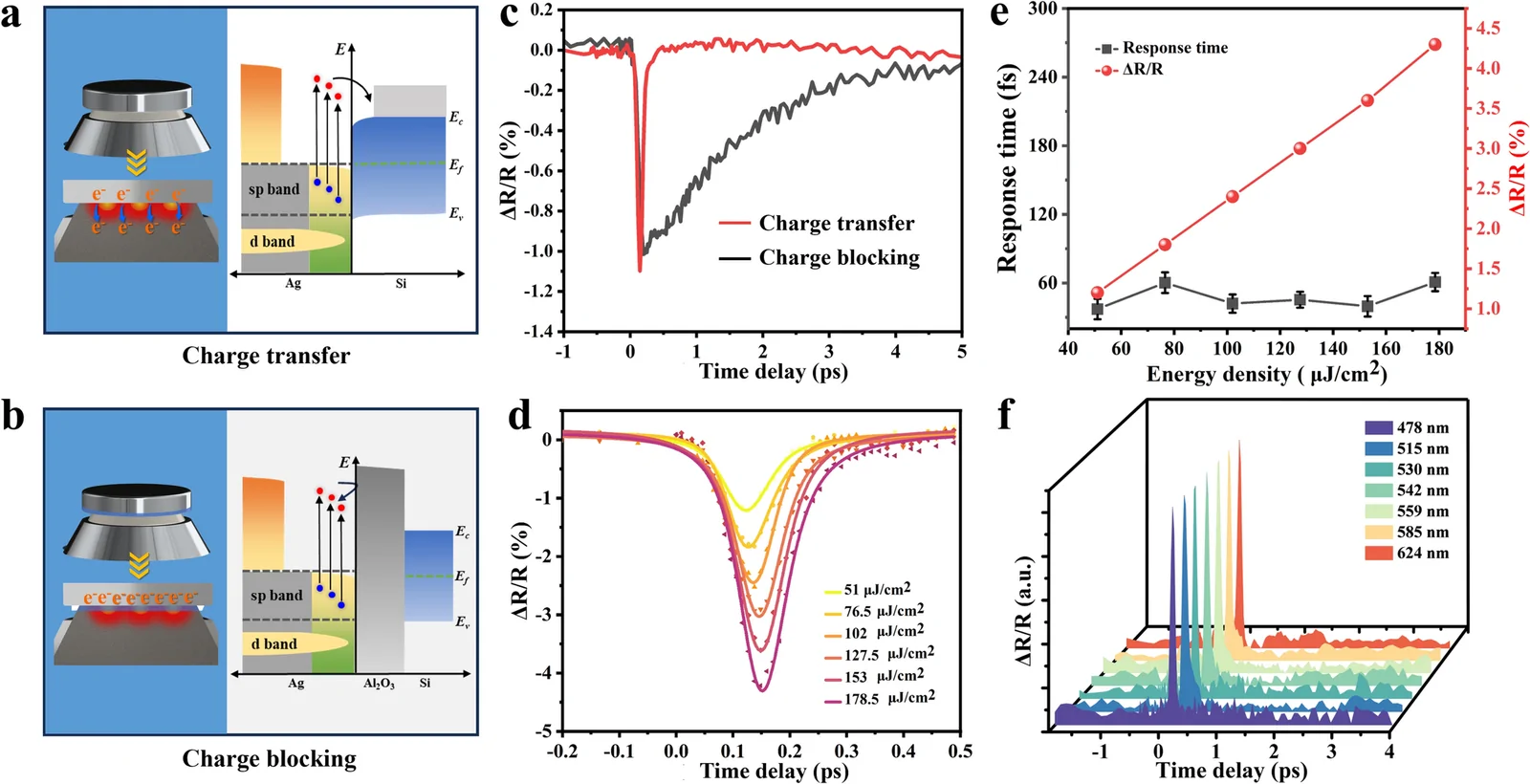
Schematic illustration of the hot electron relaxation and transient optical measurements. **a, b** Schematic illustration of the hot electron relaxation pathways in samples with and without an alumina insulating layer. **c** Transient dynamic spectra of SSDMA with and without an alumina insulating layer, excited by a 660 nm pump laser. **d** Transient dynamic spectra of SSDMA samples at different pump power densities, with a pump wavelength of 660 nm. **e** Power dependence of modulation depth and speed. Error bars show fitting uncertainties. **f** Transient dynamic spectra at various probe wavelengths, extracted from the dataset presented in Fig. 3d

To provide additional evidence supporting the hypothesis that the femtosecond all-optical modulation signal is a consequence of ultrafast hot-electron injection induced by surface plasmons at the silver-silicon interface, we designed a comparative sample incorporating a 30 nm thick aluminum oxide insulating layer. This insulating layer was precisely positioned between the silver nanodisks and the monocrystalline silicon wafer utilizing an atomic layer deposition system, with the specific purpose of impeding the transfer of hot electrons from the silver surface to the monocrystalline silicon. The schematic of the design is shown in Fig. 4a, b, illustrating the relaxation pathways of hot carriers in samples both with and without an aluminum oxide insulating layer. Figure 4c displays the transient dynamics curves obtained from ultrafast spectroscopy measurements of the silver-silicon nanodisk samples with and without the aluminum oxide insulation layer. The inclusion of the aluminum oxide layer hinders the rapid transfer of hot electrons to the conduction band of monocrystalline silicon through the silver-monocrystalline silicon interface. As a result, there is a noticeable predominance of slower component dynamics, specifically in the heat transfer process between hot electrons and phonons, which impacts the modulation speed of the sample. This observation is clearly demonstrated in the transient spectra depicted in Fig. 4c, where the rate of recovery within the 0–5 ps timeframe is significantly slower for the sample with the alumina insulating layer than for that without it. Overall, these comparative experiments provide compelling evidence that sub-100 fs modulation of SSDMA is primarily driven by the ultrafast transfer of nonequilibrium electrons at the silver-single crystal silicon interface. This transfer induces rapid changes in the interface dielectric constant, enabling sub-100 fs ultrafast modulation of the probe light.

The power dependence of the transient all-optical modulation performance of the SSDMA was further investigated, as shown in Fig. 4d. Under 660 nm excitation, the pump fluence was varied from 51 to 178.5 μJ cm^−2^. The transient responses at different pump fluences were analyzed using the Gaussian–Lorentzian deconvolution described above to extract intrinsic modulation times and their dependence on modulation depth (Fig. 4e). The data show that the all-optical modulation depth of the sample increases linearly from 1.2% to 4.3%, indicating that the ultrafast modulation signal does not stem from the nonlinear effects of silicon. Additionally, as the pump power increases, the modulation speed remains within an ultrafast timescale of less than 100 fs. To exclude potential resonance-specific artifacts, transient dynamics were further extracted at multiple probe wavelengths (Fig. 4f). Upon resonant excitation of the Ag-Si interfacial plasmon at 660 nm, Landau-damping induced interfacial carrier extraction transiently renormalizes the interfacial permittivity, causing all probe modes with near fields co-localized at this boundary to exhibit consistent sub-100 fs dynamics. These results further indicate that the sub-100 fs modulation is governed by a unified interfacial carrier dynamics mechanism.

### Ultrafast Energy-Transfer Simulation Based on Coupled Wave-Optics and Two-Temperature Modeling

To further verify the activation of the lossless ultrafast energy-transfer channel, we established a coupled multiphysics simulation framework that integrates the COMSOL Wave Optics Module with the general form of the two-temperature model (TTM). This approach enables simultaneous determination of the spatial distribution of electromagnetic absorption within the SSDMA metastructure and the subsequent temporal evolution of the electron and lattice temperatures. A full description of the numerical procedure is provided in Section S7. The electromagnetic loss density Q(x,y,z) was first obtained from the Wave Optics Module under excitation wavelengths of 660 and 400 nm. This spatially resolved loss profile was then modulated by the temporal Gaussian envelope of the pump pulse to generate a time-dependent volumetric heat source, $S(x,y,z,t)=Q(x,y,z)\hspace{0.17em}I(t)$, which serves as the excitation term in the TTM [14, 35–39]. The transient nonequilibrium dynamics were computed by solving the coupled TTM equations using the finite-element method. The electron temperature ${T}_{e}$ and lattice temperature ${T}_{l}$ evolve according to:

2a $$\begin{array}{cccc}& {C}_{e}({T}_{e})\frac{\partial {T}_{e}}{\partial t}=\nabla \hspace{0.17em}\cdot ({\kappa }_{e}\nabla {T}_{e})-{G}_{ep}({T}_{e}-{T}_{l})-{G}_{es}({T}_{e}-{T}_{s})+S(x,y,z,t),& & \end{array}$$

2b $$\begin{array}{cccc}& {C}_{l}\frac{\partial {T}_{l}}{\partial t}=\nabla \hspace{0.17em}\cdot ({\kappa }_{l}\nabla {T}_{l})+{G}_{ep}({T}_{e}-{T}_{l}),& & \end{array}$$

where ${C}_{e}=\gamma {T}_{e}$ and ${C}_{l}$ are the electron and lattice heat capacities, ${\kappa }_{e}$ and ${\kappa }_{l}$ are the corresponding thermal conductivities, ${G}_{ep}$ is the electron–phonon coupling constant of Ag, and ${G}_{es}$ is an effective volumetric electron-silicon interfacial coupling coefficient (${\mathrm{W}}\, {\mathrm{m}}^{-3} {\mathrm{K}}^{-1}$) that parameterizes ultrafast interfacial energy extraction from the Ag electron subsystem into silicon. In the present simulations, ${G}_{es}=2\times {10}^{16}$ ${\mathrm{W}}\, {\mathrm{m}}^{-3} {\mathrm{K}}^{-1}$, obtained by converting literature-reported interfacial electron-substrate conductances for metal/Si interfaces into an equivalent volumetric form using the characteristic interfacial localization length in the SSDMA geometry [40, 41], see Supplementary Materials Sect. 7 for details. In this formulation, the silicon substrate is treated as an effective thermal reservoir maintained at the ambient temperature ${T}_{s}=293$ K. This approximation reflects the fact that, on the sub-100-fs timescale resolved in the present pump-probe measurements, the dominant energy-transfer pathway is interfacial extraction from Ag electrons into a much larger silicon heat capacity. The coupling term ${G}_{es}\left({T}_{e}-{T}_{s}\right)$ therefore represents net energy diffusion from the Ag electron subsystem into a semi-infinite silicon substrate, without explicitly resolving the transient temperature evolution within silicon. This reduced description isolates the experimentally relevant interfacial energy-transfer channel while avoiding additional unconstrained degrees of freedom (see Section S7 for details).

**Fig. 5:**
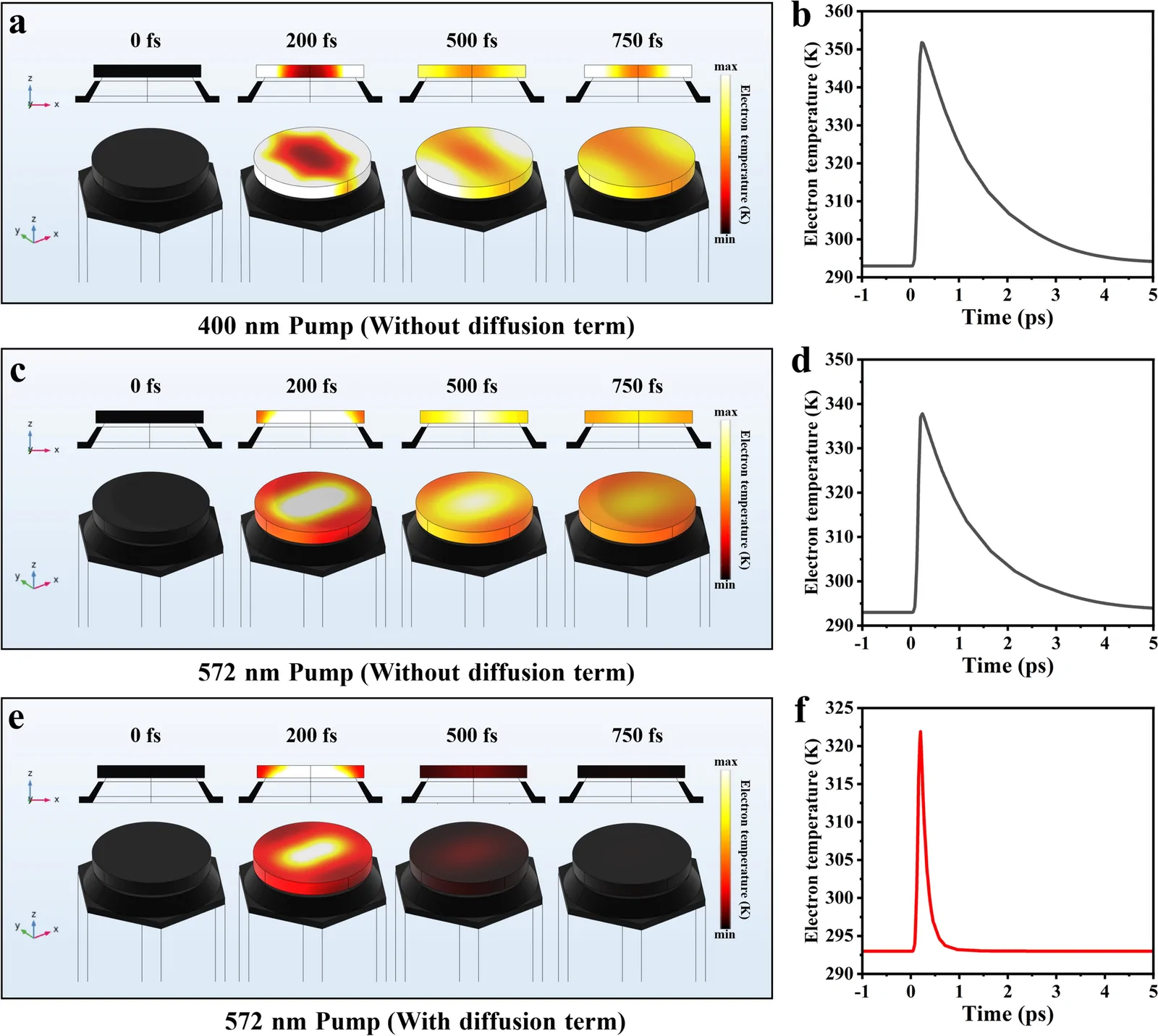
Time-resolved thermal electron temperature simulation. **a** Electron temperature of the SSDMA structure at different time points under 400 nm pump wavelength excitation. **b** Time-resolved temperature spectrum of hot electrons under 400 nm pump wavelength excitation. **c** Electron temperature of the SSDMA structure at different time points under 572 nm pump wavelength excitation. **d** Time-resolved temperature spectrum of hot electrons under 572 nm pump wavelength excitation. **e** Electron temperature of the SSDMA structure at different time points under 572 nm pump wavelength excitation, including the diffusion term. **f** Time-resolved temperature spectrum of hot electrons, including the diffusion term, under 572 nm pump wavelength excitation

Figure 5a–d presents the evolution of the electronic temperature under 660 and 400 nm excitation during the first 5 ps. The results reveal that the electron subsystem within the silver nanostructure remains at an elevated temperature even at approximately 750 fs when only intrinsic electron–phonon coupling is considered, indicating that conventional plasmonic relaxation pathways intrinsically limit the attainable modulation speed. To overcome this bottleneck, the interfacial coupling term ${G}_{es}({T}_{e}-{T}_{s})$ was incorporated to account for the ultrafast transfer of hot electrons into the silicon substrate. As shown in Fig. 5e, f, inclusion of this interfacial channel substantially accelerates the cooling of the electron subsystem and yields dynamics that faithfully reproduce the experimentally observed sub-100 fs response. The simulations performed under 660 nm excitation also exhibit excellent agreement with the experimental transient results, and full datasets are provided in Fig. S6.

## Conclusions

In summary, we demonstrated a metastructured plasmonic antenna capable of experimentally resolved sub-100 fs all-optical modulation, which surpasses the electron–phonon relaxation bottleneck that limits conventional plasmonic devices. By engineering the spatial confinement of interfacial plasmonic modes, a nonthermal hot-electron extraction pathway is activated that competes with electronic thermalization and precedes substantial lattice heating. This interface-governed ultrafast energy-transfer regime is quantitatively supported by femtosecond transient spectroscopy and a coupled electromagnetic-thermal physical model, establishing a clear mechanistic origin for the observed sub-100 fs dynamics. More broadly, this work identifies interfacial carrier extraction as an effective strategy for overcoming intrinsic electron–phonon-limited temporal constraints in plasmonic nanostructures and provides a physically grounded framework for the design of ultrafast optoelectronic components operating near intrinsic carrier-dynamics limits.

## Supplementary Information

Below is the link to the electronic supplementary material.

Supplementary file1 (DOCX 14262 KB)
