# Supplementary material for: Sub-100 Femtosecond All-Optical Modulation Beyond Electron–Phonon Limits
Source: Nanomicro Lett. 2026 Apr 7;18:315. doi: 10.1007/s40820-026-02166-z (PMC13057177; doi:10.1007/s40820-026-02166-z)
Supplement: Supplementary file 1 — Supplementary file1 (DOCX 14262 KB) [file 40820_2026_2166_MOESM1_ESM.docx]

Supporting Information for

**Sub-100 Femtosecond All-Optical** **Modulation Beyond Electron-Phonon Limits**

Renxian Gao^1,2#^, Jiayu Li^3#^, Xiaoxiang Dong^1#^, Yonglin He^1^, Wenbin Chen^3^, Peiwen Ren^1^, Xiaoyu Zhao^2^, Ming-De Li^4*^, Zhilin Yang^1*^

^1^ College of Physical Science and Technology, Xiamen University, Xiamen 361005, P. R. China

^2^ College of Materials and Environmental Engineering, Hangzhou Dianzi University, Hangzhou 310018, P. R. China

^3^ Key Laboratory for Preparation and Application of Ordered Structural Materials of Guangdong Province, Department of Chemistry, Shantou University, Shantou 515063, P. R. China

^4^ School of Physics and Optoelectronic Engineering & Guangdong Provincial Key Laboratory of Sensing Physics and System Integration Applications, Guangdong University of Technology, Guangzhou, Guangdong 510006, P. R. China

^#^Renxian Gao, Jiayu Li, and Xiaoxiang Dong contributed equally to this work.

*Corresponding authors. E-mail: [zlyang@xmu.edu.cn](mailto:zlyang@xmu.edu.cn) (Zhilin Yang); [mdli@stu.edu.cn](mailto:mdli@stu.edu.cn) (Ming-De Li)

**S1 Fabrication of silver-single crystal silicon nanodisk metastructure antenna（SSDMA）**


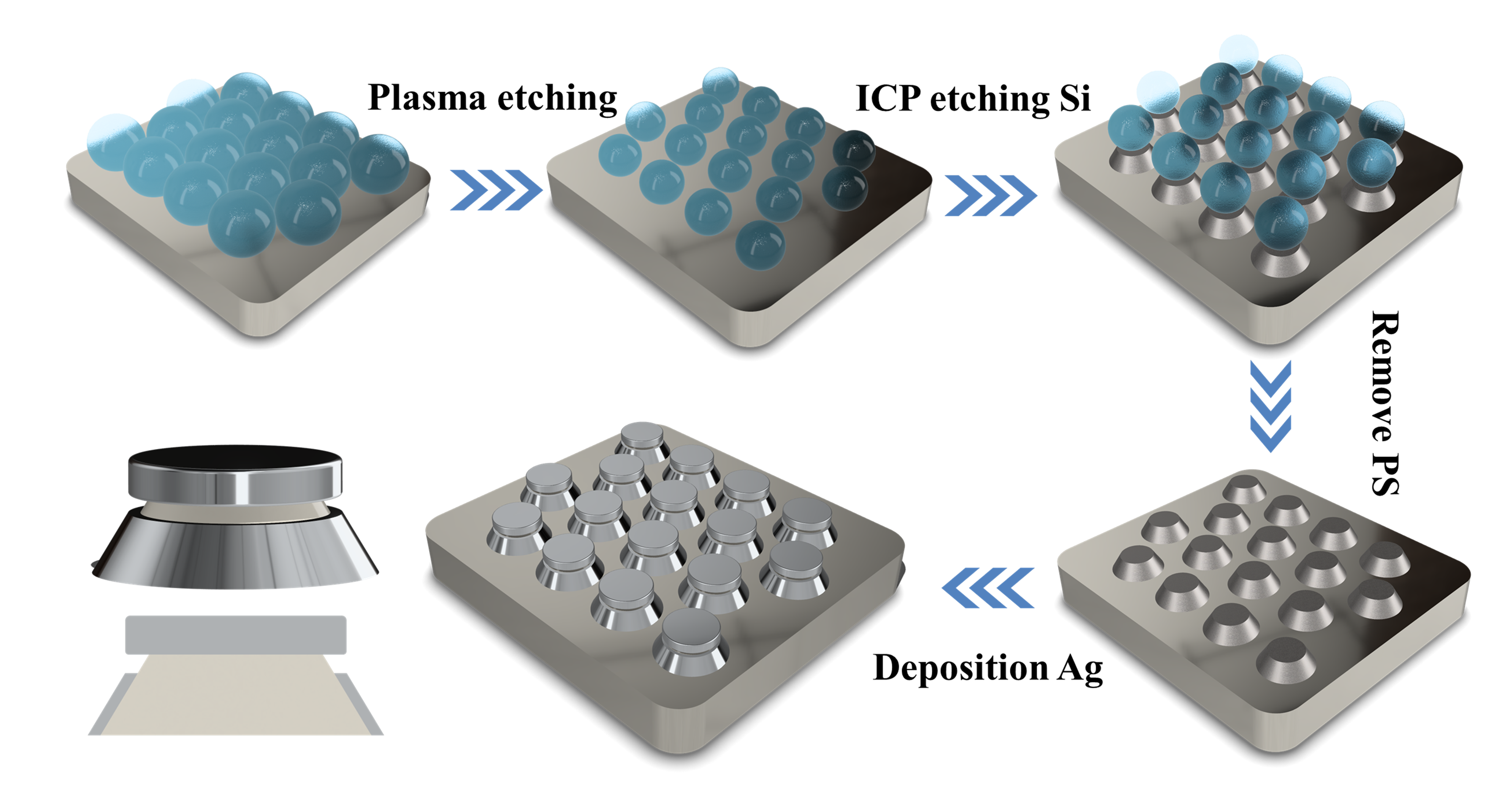


**Fig. S1** The process flow of the SSDMA

**S2** **Scanning electron microscope image of SSDMA**

The morphology of the samples was characterized using Scanning Electron Microscopy (SEM). Fig. S2a reveals a regularly arranged, hexagonally close-packed periodic array of nanopillars with a 300 nm diameter of the silver nanodisks and a periodicity of 500 nm. Low-magnification SEM images of the sample, as displayed in Fig. S2b, demonstrate a high degree of uniformity over a large spatial extent.


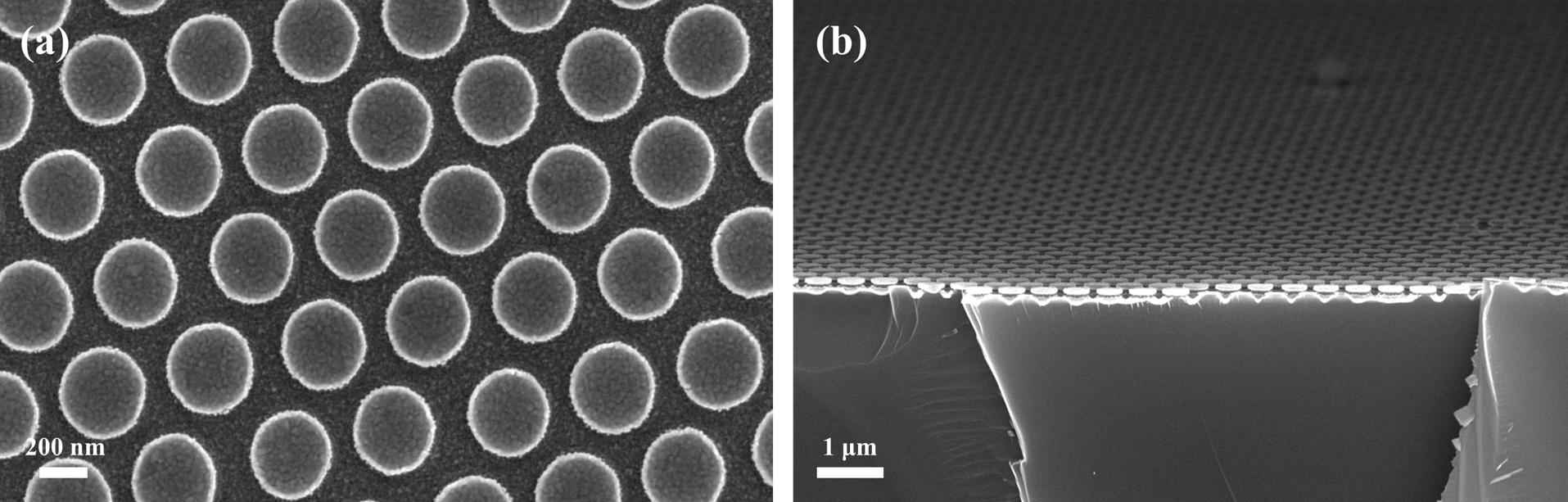


**Fig. S2** SEM image of SSDMA. **a** Top-view SEM image of the sample. **b** Side-view low-magnification SEM image of the sample

**S3 Numerical simulations of the SSDMA and pure silver nanoparticle array**

To validate the rationality of the designed silver-single crystal silicon interface localized optical field metastructure, numerical simulations were performed. We compared the plasmonic electric field intensity at the silver-silicon interface between the superstructures and pure silver nanodisk arrays. The numerical simulation results from Fig. S3 indicate that the plasmonic photoelectric field intensity at the silver-silicon interface for Structure 2 is substantially higher than that of Structure 1. Consequently, the localized photoelectric field in Structure 2 is stronger, which suggests a potential advantage of this structure in inducing efficient hot electron transfer and facilitating ultrafast all-optical modulation through surface plasmon excitation. The finite element calculated reflectance spectra in Fig. S3b demonstrate that, compared to Structure 1, Structure 2 exhibits more pronounced resonance absorption dips at 521 nm, 570 nm, and 687 nm. This suggests that the SSDMA has a superior capability for multi-wavelength ultrafast all-optical modulation compared to traditional silver nanodisk arrays.


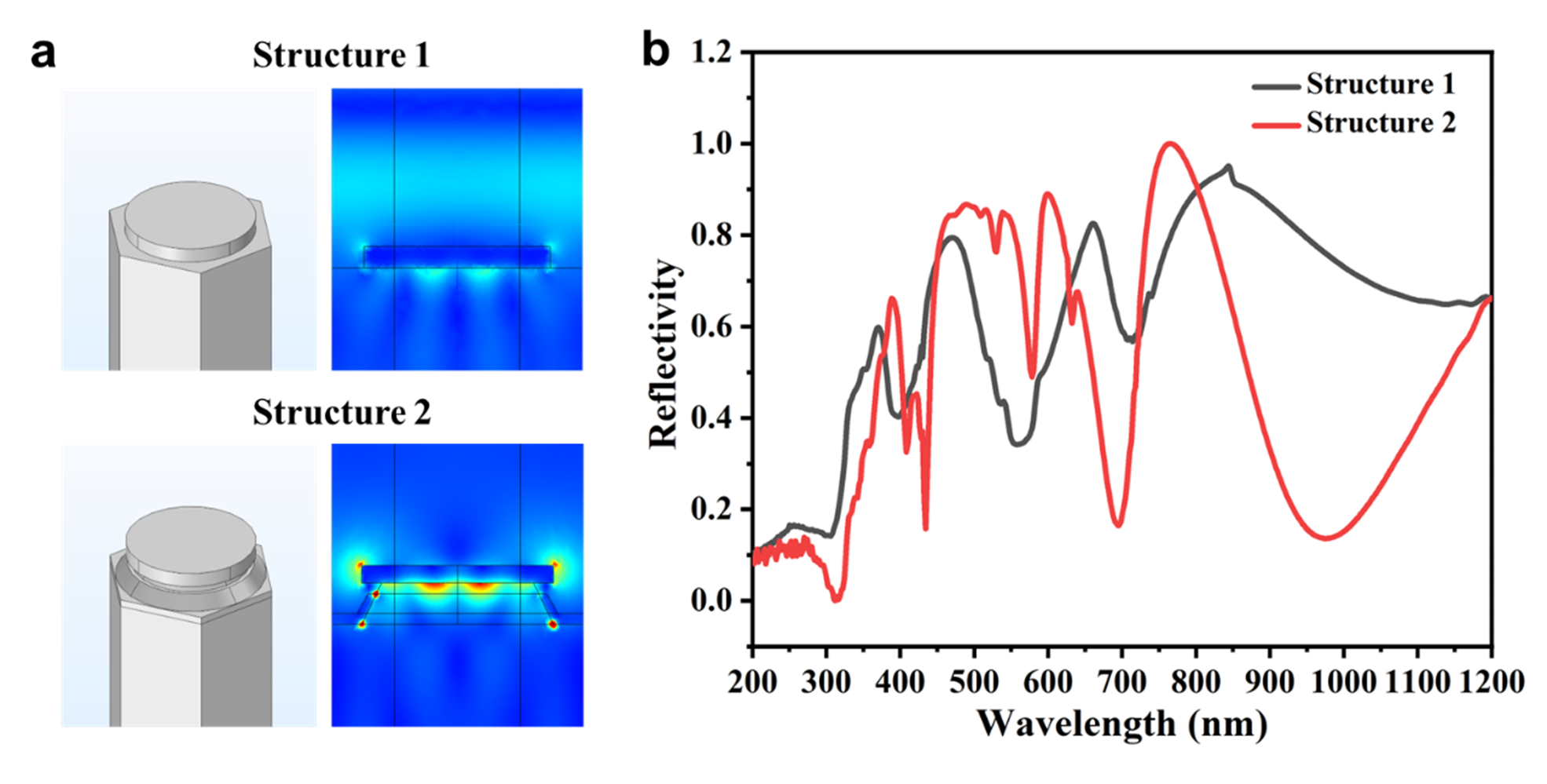


**Fig. S3** Comparative numerical simulations of the SSDMA and pure silver nanoparticle array. **a** Numerical simulation of the electric field distribution for both the SSDMA and pure silver nanoparticle arrays. **b** Numerical simulation results of the reflection spectra for the two structures

**S4 Schematic diagram of the pump-probe experiment**


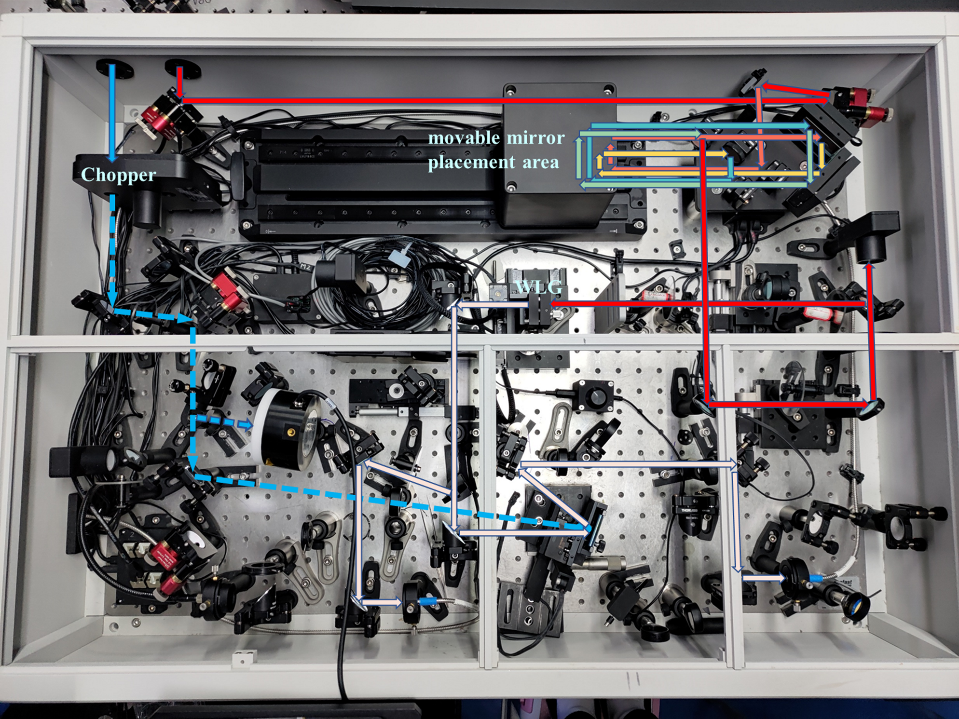


**Fig. S4** Schematic diagram of the pump-probe experiment

**S5 Transient dynamic spectral lines with experimental error bars**

**
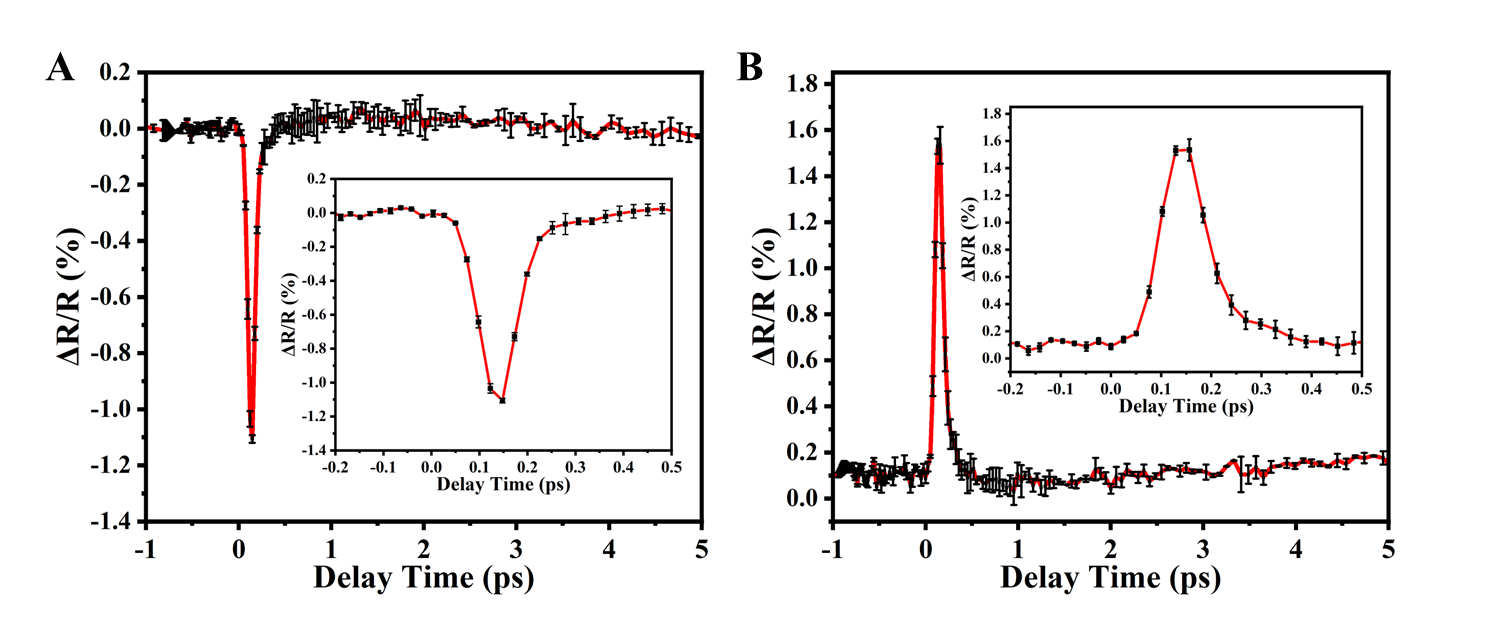
**

**Fig. S5** Transient dynamic spectral lines with experimental error bars. **a** Transient dynamic spectral lines with a pump wavelength of 660 nm and a probe wavelength of 559 nm, with experimental error bars. **b** Transient dynamic spectral lines with a pump wavelength of 572 nm and a probe wavelength of 589 nm, with experimental error bars

**S6 Transient spectroscopy data fitting method**

The transient spectroscopy data were analyzed using a convolution-based fitting procedure, in which the measured kinetics are modeled as the convolution of the instrument response function (IRF) and the intrinsic relaxation dynamics of the sample. The IRF is represented by a Gaussian function, while the material’s intrinsic response is described by a single-exponential decay function. The fitting procedure follows the equation:

$$y=y_{0}+\left(f_{1}*f_{2}\right)\left(x\right)$$

Where $f_{1}(x)$ is the IRF, represented by a Gaussian function:

$$f_{1}(x)=\sqrt{\frac{4\ln2}{\pi}}\cdot\frac{e^{-\frac{4\ln2}{w_{G}^{2}}x^{2}}}{w_{G}}$$

and$f_{2}(x)$is the optical response of the material, modeled by a Lorentzian function:

$$f_{2}(x)=\frac{4A}{\pi(w_{LR}+w_{LI})}\cdot\left(\frac{1}{1+\frac{4(x-x_{c})^{2}}{w_{LI}^{2}}}\right)$$

The parameters of the Lorentzian function, including the scaling factor *A*, the widths and *w_LR_ and w_LI_*, and the characteristic value *x_c_*, were optimized through the deconvolution process to extract the material's intrinsic optical response.

We performed transient spectral fitting for the data corresponding to Fig. 2g and 2h, following the convolution-based model described above. The IRF was modeled as a Gaussian function with a full width at half maximum (*FWHM*) set to 1.2 times the *FWHM* of the laser pulse (84 fs), accounting for pulse broadening effects induced by multiple reflections and beam path dispersion in the optical setup. This yields an IRF *FWHM* of 100.8 fs, corresponding to a standard deviation of σ=FWHM/[2√(2*ln2)]=42.8 fs, which defines the width parameter *w_G_* of the Gaussian IRF function. In the fitting process, the material's intrinsic optical response was assumed to follow a Lorentzian function, characterized by its standard deviation *w_Lr_*, from which the Lorentzian *FWHM* was calculated as *FWHM*=2·*w_Lr_*. The intrinsic signal response time was subsequently obtained by subtracting the IRF *FWHM* from the Lorentzian *FWHM*, thereby yielding a corrected estimation of the relaxation time corresponding to the optical response of the sample. The extracted relaxation times were 37±9 fs for Figure 2g and 66.7±14 fs for Figure 2h. The fitting quality, evaluated by the coefficient of determination (R²), was 0.99316 and 0.98805 for Fig. 2g, h, respectively, demonstrating a high level of agreement between the experimental data and the convolution-based model. The full set of optimized fitting parameters, including amplitude coefficients, central positions, and spectral linewidths, is provided in Tables S1 and S2.

**Table S1** Fitting Parameters Extracted from Fig. 2g


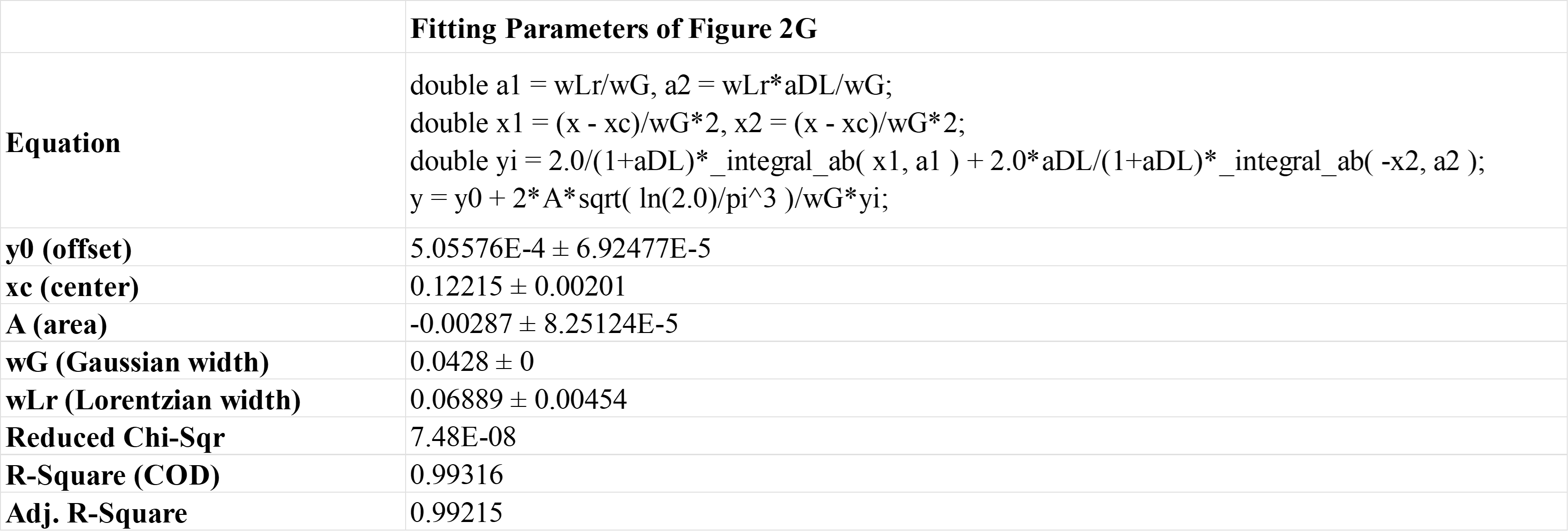


**Table S2** Fitting Parameters Extracted from Fig. 2h


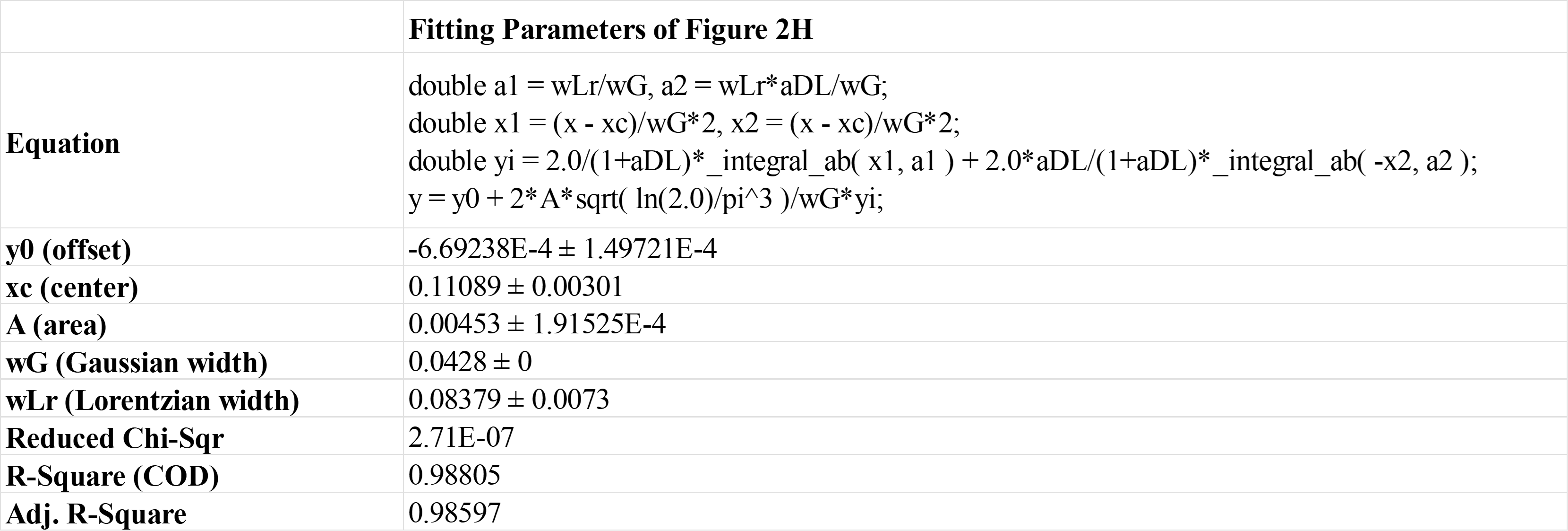


**S7 Coupled simulation of wave optics and two-temperature model**

A self-consistent multiphysics model was constructed to describe the interaction between femtosecond optical excitation and the nonequilibrium carrier dynamics occurring within the SSDMA metastructure. The formulation combines the electromagnetic field distribution obtained from COMSOL’s Wave Optics Module with a generalized two-temperature model that captures both the ultrafast electron heating following optical absorption and the subsequent exchange of energy between electrons, phonons, and the silicon substrate.

The electromagnetic response was first computed by solving the frequency-domain Maxwell equation under the experimental excitation wavelength. From the resulting field distribution, the volumetric electromagnetic loss density was evaluated as

$$Q(x,y,z)=\frac{1}{2}\omega\varepsilon_{0}\varepsilon^{''}\mid E(x,y,z)\mid^{2},$$

which represents the local rate of optical energy deposition into the electron subsystem. To incorporate the femtosecond temporal profile of the excitation, this loss density was multiplied by the temporal Gaussian pump envelope to form the heat source term $S(x,y,z,t)=Q(x,y,z)I(t)$. This term serves as the energy input driving the nonequilibrium electron temperature in the two-temperature model, and its unit of W·m^-3^ ensures direct compatibility with the volumetric nature of the governing equations.

The evolution of the electron temperature $T_{e}$and lattice temperature $T_{l}$was obtained by solving the coupled two-temperature equations:

$$\begin{matrix} & C_{e}(T_{e})\frac{\partial T_{e}}{\partial t}=\nabla\text{ }\cdot(\kappa_{e}\nabla T_{e})-G_{ep}(T_{e}-T_{l})-G_{es}(T_{e}-T_{s})+S(x,y,z,t), & & \end{matrix}$$

$$\begin{matrix} & C_{l}\frac{\partial T_{l}}{\partial t}=\nabla\text{ }\cdot(\kappa_{l}\nabla T_{l})+G_{ep}(T_{e}-T_{l}). & & \end{matrix}$$

In this formulation, $C_{e}=\gamma T_{e}$denotes the electron heat capacity, $\kappa_{e}=\kappa_{0}T_{e}/T_{l}$the electron thermal conductivity, and $C_{l}$and $\kappa_{l}$the corresponding lattice quantities. The term $G_{ep}$describes the electron–phonon coupling within silver. All quantities were assigned experimentally reported or literature-validated values, including $\gamma=71\text{ }{\text{J}\text{/}\text{m}}^{-3}\text{K}^{-2}$, $\kappa_{0}=317 {\text{W}\text{/}\text{m}}^{-1}\text{K}^{-1}$, $C_{l}=2.492\times{10}^{6}\text{ }{\text{J}\text{/}}^{-3}\text{K}^{-1}$, $\kappa_{l}=2.6{\text{W}\text{/}\text{m}}^{-1}\text{K}^{-1}$, and $G_{ep}=2.2\times{10}^{16}\text{ }{\text{W}\text{/}\text{m}}^{-3}\text{K}^{-1}$. The temperature field $T_{s}(x,y,z,t)$represents the local lattice temperature of the single-crystal silicon at the metal–semiconductor interface, calculated independently from the heat-diffusion equation within the silicon region. A key element of the model is the additional interfacial energy-transfer term $G_{es}(T_{e}-T_{s})$introduced into Eq. (S1). This term captures the rapid transfer of nonequilibrium hot electrons from the silver nanostructure into the single-crystal silicon substrate and is formulated as an effective volumetric energy-sink channel. In the present simulations, the interfacial coupling coefficient was set to $G_{es}=2.0\times{10}^{16}\text{ }W\text{ }m^{-3}\text{ }K^{-1}.$ This value was estimated by converting reported metal–Si interfacial electron–substrate conductance values ($\sigma_{e}\sim{10}^{7}\text{–}{10}^{8}\text{ }W\text{ }m^{-2}\text{ }K^{-1}$) into an equivalent volumetric coefficient using $G_{es}\approx\sigma_{e}/d_{\mathrm{eff}}$, where $d_{\mathrm{eff}}\approx5$nm represents the nanoscale region over which plasmonic absorption and nonequilibrium electrons are localized near the Ag–Si interface in the SSDMA geometry. The coupled electromagnetic–thermal equations were solved using the finite-element method with refined spatial meshing at the Ag–Si interface to accurately resolve steep thermal gradients. Time steps on the order of sub-femtoseconds were employed to capture the rapid electron heating and cooling dynamics. The resulting simulations provide a quantitative and physically grounded description of hot-electron generation, interfacial transport, and ultrafast energy extraction within the metasurface, forming the theoretical basis for the lossless modulation channel observed experimentally.

**S8 Time-resolved electron temperature distribution of the SSDMA**


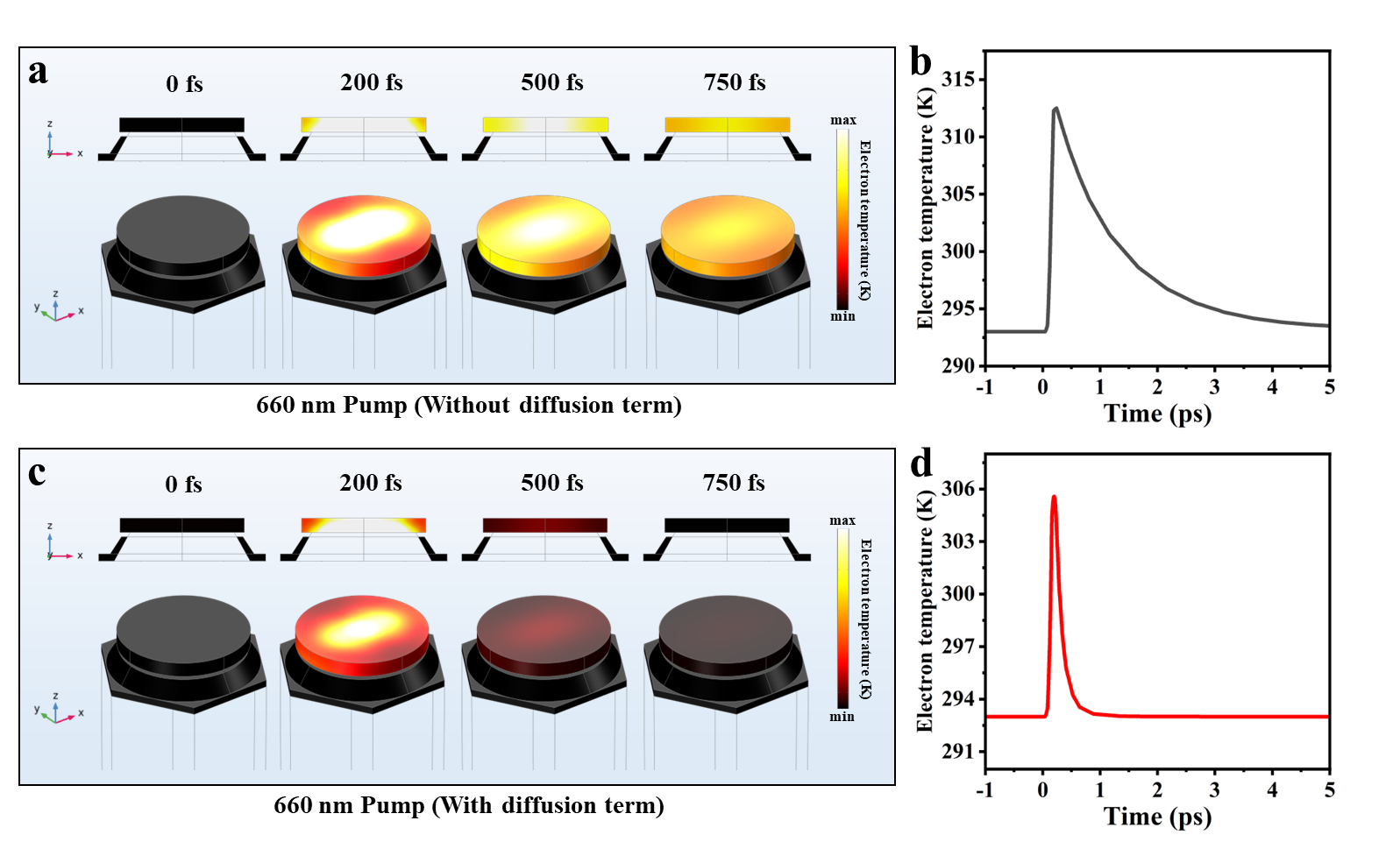


**Fig. S6** Time-resolved electron temperature distribution. **a** Electron temperature of the SSDMA structure at different time points under 660 nm pump wavelength excitation. **b** Time-resolved temperature spectrum of hot electrons under 660 nm pump wavelength excitation. **c** Electron temperature of the SSDMA structure at different time points under 660 nm pump wavelength excitation, including the diffusion term. **d** Time-resolved temperature spectrum of hot electrons, including the diffusion term, under 660 nm pump wavelength excitation
